# Dynamic Longitudinal Associations Between Social Support and Cognitive Function: A Prospective Investigation of the Directionality of Associations

**DOI:** 10.1093/geronb/gbw135

**Published:** 2016-11-01

**Authors:** Jing Liao, Graciela Muniz-Terrera, Jenny Head, Eric John Brunner

**Affiliations:** Department of Epidemiology and Public Health, UCL, London, UK

**Keywords:** Cognitive aging, Longitudinal change, Reciprocal, Social support, Temporality

## Abstract

**Objective:**

To investigate the reciprocity of social support and cognitive function in late life.

**Method:**

Analyses were based on three parallel repeat measures of social support and cognition from the Whitehall II cohort, providing 10-year follow-up of 6,863 participants (mean age 55.8 years, *SD* 6.0 at baseline). Alternative hypotheses were evaluated via four bivariate dual change score models: Full coupling model estimated mutual influences of social support and cognition on subsequent changes in each other; social causation model assumed a unidirectional influence from social support onto changes in cognition, while the opposite assumption was tested by health selection model; last, no coupling model suggested independent growth of these two sets of variables.

**Results:**

A better cognition at the preceding stage was related to less positive changes in confiding support and less negative changes in practical support. In contrast, influences from social support on subsequent changes in cognition were not detected.

**Discussion:**

This empirical study provides some evidence for the health selection mechanism, such that cognition modified changes in social support. The hypothesized neuroprotective effect of social support was not detectable.

Prospective studies suggest social relationships confer cognitive benefits in late life (Fratiglioni, Wang, Ericsson, Maytan, & Winblad, 2000; Seeman et al., 2010). Cognitive decline, however, is likely to curtail social engagement (Shouse, Rowe, & Mast, 2013) and strain social relationships (Lang, 2001). Consequently, the observed neuroprotective effect of social relationships may be produced by reverse causality (Stoykova, Matharan, Dartigues, & Amieva, 2011), insofar as cognitive decline may be the cause rather than the consequence of the lack or deterioration of social relationships.

Social causation and health selection are two major hypotheses widely used to understand the dynamic associations of social relationships and cognition (Mackinnon, Christensen, Hofer, Korten, & Jorm, 2003). Social causation hypothesizes that better social support (Seeman, Lusignolo, Albert, & Berkman, 2001) and larger social networks (Béland, Zunzunegui, Alvarado, Otero, & Del Ser, 2005) retard cognitive decline, aligned with social integration theory reviewed by Berkman, Glass, Brissette, and Seeman (2000). Conversely, health selection operates if cognitive limitations affect quantity (Aartsen, van Tilburg, Smits, & Knipscheer, 2004) and quality (Gurung, Taylor, & Seeman, 2003) of social relationships, due to reduced social functioning (Washburn, Sands, & Walton, 2003) and diminished social reciprocity (Lang, Wagner, & Neyer, 2009). The direction of health selection effect is unclear. Poor cognition may hamper social integration or instead may mobilize support and consolidate social network (van Tilburg & Broese van Groenou, 2002).

The dynamic interplay between social relationships and cognition has been studied in only three population-based longitudinal aging cohorts. Thomas (2011) investigated the cross-lagged associations between social engagement and cognitive limitation, showing social causation in women and health selection in men. Using the same statistical technique, Li and Zhang (2015) found bidirectional associations of diverse social networks and cognitive maintenance in a Chinese elderly sample. Ellwardt and colleagues (2013) modeled directional parallel latent growth curves (PLCM) of social support and cognition in the Longitudinal Ageing Study Amsterdam and found evidence for social causation only.

Cross-lagged and directional PLCM models address the possibility of reciprocity, but they have weaknesses. The cross-lagged model estimates each variable as a combined function of this variable’s preceding value, the other variable at the previous time and a time-specific residual. Differences in measurement reliability of the variables considered may bias the estimation (Rogosa, 1980), and this model may overlook systematic growth across multiple waves (Bollen & Curran, 2006). The directional PLCM regresses the latent slope parameter of one repeat measure onto the intercept of the other, assuming the intercept is the initial time point when change starts. This assumption depends on the intercept placement (Grimm, 2007) and may be inappropriate in most aging research (Robitaille, Muniz, Piccinin, Johansson, & Hofer, 2012). The dual change score model (DCSM; McArdle & Hamagami, 2001) is an alternative approach that accounts for different reliabilities (i.e., amount of measurement errors) and stability (i.e., amount of interindividual change) in multivariate longitudinal data and simultaneously estimates intervariable lead-lag effects and intravariable growth patterns. Furthermore, DCSM allows statistical comparison of alternative hypotheses concerning the time-lagged associations.

The current study employs bivariate DCSM (BDCSM) to investigate bidirectional relationships between three repeat measures of social support and cognition in the Whitehall II cohort, enabling rigorous evaluation of social causation and health selection processes. Given that high-quality social relationships may be more important than social network size in preserving cognitive abilities (Amieva et al., 2010), we specifically assessed types of support transmitted (i.e., confiding support, practical support) and supportive role fulfillment (i.e., negative aspects of relationships) by close social ties that provide the most reliable (Aartsen et al., 2004) and emotionally rewarding (Carstensen, Fung, & Charles, 2003) support as people age. Confiding support includes provision of empathy and information, whereas practical support involves tangible aid and helping behaviors (Gottlieb & Bergen, 2010). Conversely, well-intentioned support may elicit social strain if the recipient finds support is unsuitable, intrusive, or overcontrolling (Rook, 1984). Two domains of cognitive function, namely executive function and memory, were investigated in view of the cognitive-domain-specific associations reported previously (Gow, Corley, Starr, & Deary, 2013; Liao et al., 2014).

## Method

### Study Population

The Whitehall II cohort recruited 10,308 participants from 20 London based civil service departments in 1985–1988. At study baseline, all participants underwent clinical health check-ups and completed self-administrated questionnaires. Subsequent data collection alternated between postal questionnaires alone and postal questionnaires accompanied by clinical check-ups (Marmot & Brunner, 2005). The current study used the parallel repeat measures of cognition and social support over a 10-year period at Phases 5 (1997–1999), 7 (2002–2004), and 9 (2007–2009). The University College London Medical School Committee on ethics of human research approved the Whitehall II study.

### Cognitive Function

Executive function was derived from three tests: the Alice Heim 4-I test (AH4-I), an inductive reasoning test, consisting of 65 verbal and numeric items to be completed within 10 min, and two tests of verbal fluency, phonemic fluency and semantic fluency, where participants were instructed to recall in writing as many words beginning with “S” (phonemic) and as many animal names (semantic) as possible in 1 min. The verbal fluency tests involve both linguistic (storage and retrieval) and fluent (speed and efficiency) components. Despite their knowledge-based nature, the verbal fluency tests are widely used to measure executive function (Bryan & Luszcz, 2000). A composite score of executive function was created by first standardizing the raw scores of these three tests into *Z*-scores (mean = 0; *SD* = 1) based on Phase 5 mean and *SD*; then these *Z*-scores were averaged to yield executive function scores. Confirmatory factor analysis supports the construction of the composite executive function score, with the factor loadings of 0.69 for AH4-I, 0.71 for phonemic fluency, and 0.84 for semantic fluency.

Short-term verbal memory was measured by a 20-word audiotaped list of single- or double-syllable words presented at 2-s intervals, which participants were required to recall in writing over 2 min. Included cognitive tests had high test–retest reliability (0.67–0.89) within 3 months (Singh-Manoux et al., 2012).

### Social Support

Social support was measured by the Close Persons Questionnaire, which had good test–retest reliabilities over a 4-week interval (0.71–0.88; Stansfeld & Marmot, 1992). Factor analysis identified three subscales (Stansfeld & Marmot, 1992): confiding support, practical support, and negative aspects of close relationships. Confiding support (seven items, Cronbach’s α = 0.86), referring to confiding and emotional support, included being given information and guidance, wanting to confide, boosting self-esteem, and sharing interest. Practical support (three items, Cronbach’s α = 0.80) indicated tangible help received. Negative aspects of close relationships (four items, Cronbach’s α = 0.65) captured adverse interactions and inadequate support. Repeated measures of social support were scaled to *Z*-scores analogous to that of cognition, where means and *SD*s at Phase 5 were used as referents.

### Covariates

Age, sex, ethnicity, longstanding illness, depressive symptoms, and prevalent chronic diseases including coronary heart disease, stroke, diabetes, or cancer measured at Phase 5 were used. Education and employment grades (i.e., clerical or support grades [low], professional or executive grades [medium], and administrative grades [high]), as indicators for socioeconomic position, were adjusted for given their positive association with social support (Ajrouch, Blandon, & Antonucci, 2005) and cognition (Singh-Manoux, Richards, & Marmot, 2005). To control for the potential effect of life events on changes in the identity of the closest person, marital history from Phase 5 to 9 was included with five categories, always married (referent), always unmarried (always single, divorced, or widowed), became unmarried (married at Phase 5 and became single, divorced, or widowed in following wave(s)), remarried (divorced or widowed at Phase 5 and remarried in subsequent wave(s)), and intermittent patterns (moved in and out of marriage more than once during the follow-up).

### Statistical Procedures

**Figure 1.**
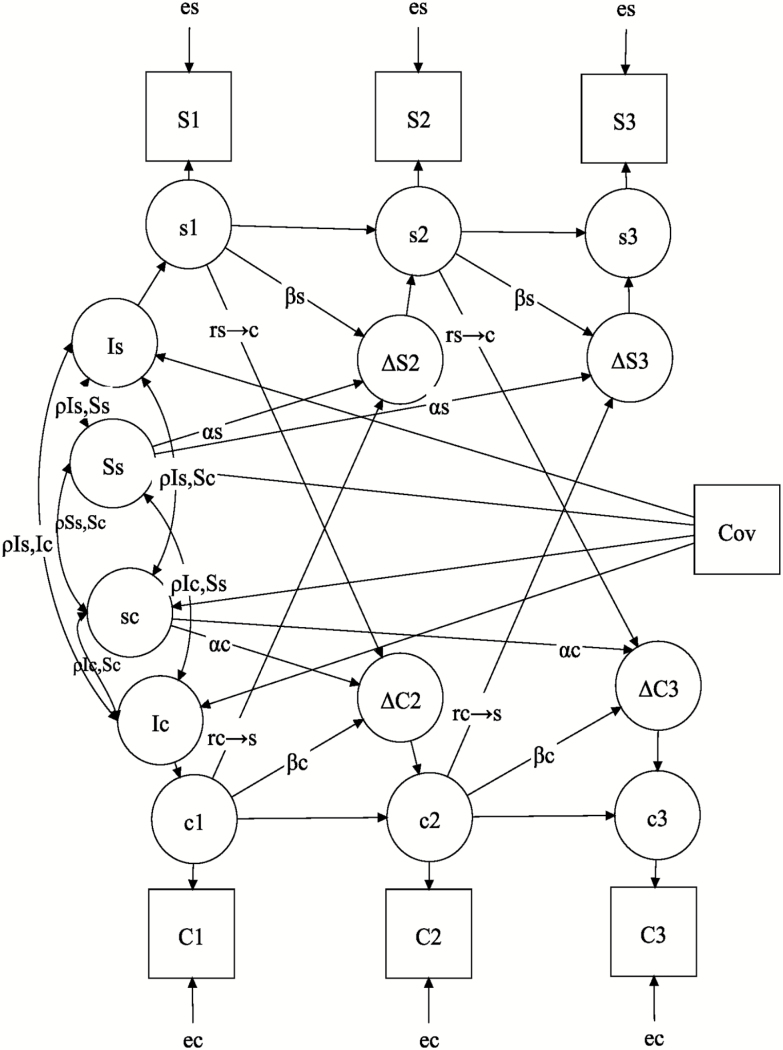
Path diagram of a conditional bivariate dual change score model (BDCSM). Observed social relationships (*S*_1–3_) and cognition (*C*_1–3_) are presented by squares, with their corresponding latent true scores (*s*_1–3_, *c*_1–3_) presented by circles. Time-invariant errors are *e*_*s*_, *e*_*c*_. Δ*S*_*t*_ and Δ*C*_*t*_ are error-free latent changes at time *t*. Intercepts (*I*_*s*_, *I*_*c*_) are anchored at Time 1, representing the reliable proportion of variance at Time 1. Overall change factors (slope: *S*_*s*_, *S*_*c*_) indicate the common constant linear component of change scores. Regression pathways are represented by one-headed arrows and variance and covariances by two-headed arrows. Unlabeled pathways are fixed to 1, except for the regression paths with the covariates (Cov). α = constant change component, β = autoproportion, and $r_{c\to s}\text{or }r_{s\to c}$ = coupling parameter.

BDCSM was used to investigate the bidirectional time-lagged relationships between social support and cognitive measure. Figure 1 depicts a path diagram of BDCSM applied to the three repeated measurements of social support (*S*_1–3_) and cognition (*C*_1–3_) assessed at Phases 5, 7, and 9 with 5-year intervals. Altogether six separate sets of analyses were carried out (2 cognitive measures × 3 measures of social support).

Under the classical true score theory, each repeated measure at time point *t* for individual *n* can be decomposed into a latent true score plus an independent time-invariant measurement error (e.g., *S*_(*t*)*n*_ = *s*_(*t*)*n*_ + *e*_*n*_; McArdle, 2009). Latent changes over 5 years between two adjacent measures of social support were labeled as Δ*S* (*s*_(*t*)_ − *s*_(*t* − 1)_) and cognition as Δ*C* (*c*_(*t*)_ − *c*_(*t* − 1)_), which were the reliable changes measured in *SD* units for their corresponding measures separated from errors ($e_{s},e_{c}$; McArdle, 2009). The latent change score in one variable was defined by three additive components: (a) the constant regression α, usually constrained to be one, referring to the latent linear slope (*S*_*s*_, *S*_*c*_); (b) the autoproportion β, proportional to the variable’s preceding value; and (c) the coupling effect γ, indicating the time-dependent effect of one variable on subsequent change in the other. Latent change scores of social support (*s*) and cognitive function (*c*) at time *t* for individual *n* can be written as,

$$\Delta S{\left(t\right)}_{n}=\;\alpha_{s}\times s_{sn}+\;\beta_{s}\times s{\left(t-1\right)}_{n}+\;r_{c\to s}\;\times \;c{\left(t-1\right)}_{n}$$

And

$$\Delta C{\left(t\right)}_{n}=\;\alpha_{c}\times s_{cn}+\;\beta_{c}\times c{\left(t-1\right)}_{n}+\;r_{s\to c}\;\times \;s{\left(t-1\right)}_{n}$$

The substantive interpretation of the coupling parameter γ was evaluated via fitting four models comparing alternative hypotheses: (a) full coupling model, assuming bidirectional influences between social support and cognition; (b) social causation model, assuming unidirectional influence from social support to latent changes in cognition $(r_{c\to s}$ = 0); (c) health selection model, assuming unidirectional influence from cognition onto latent changes in social support $(r_{s\to c}$ = 0); and (d) no coupling model, assuming independent latent changes between social support and cognition (both γ’s = 0). Compared with the full coupling model, a significant loss in goodness-of-fit for alternative nested models suggested the necessity to allow bidirectional coupling effects between social support and cognition. If there was no significant loss in goodness-of-fit, the more parsimonious model was chosen.

Multiple fit indices were used to assess the goodness-of-fit. Log-likelihood ratio test and Bayesian information criterion compared model fit between nested models. The Tucker–Lewis index (TLI), the comparative fit index (CFI), and the root mean square error of approximation (RMSEA) indicate the fit of the hypothesized models with the observed data. Cutoff values close to 0.95 were used to determine a good fit for TLI and CFI, and ≤0.06 for RMSEA (Ferrer & McArdle, 2010).

All BDCSMs controlled forage and sex on the intercepts and slopes to avoid spurious associations. The best fitting BDCSM were then controlled for other time-invariant covariates and marital history over follow-up. Supplementary analyses were conducted restricted to a subsample with complete data for social support and cognition at all three waves and among those who remained married and consistently nominated their spouse as the closest person. We also reanalyzed the data stratifying by sex and age (by mean age 55 years of Phase 5), considering previous sex-specific (Thomas, 2011) and age-group-specific (Ellwardt et al., 2013) findings.

Models were estimated by Mplus v7 (Muthén & Muthén, 2012). Missing data were handled with the full information maximum likelihood procedure, which uses both partially and fully complete cases to estimate parameters under the assumption of missing at random (Enders & Bandalos, 2001). Robust maximum likelihood estimation was used to provide corrected standard errors adjusted for non-normality of the data.

## Results

Analyses were based on 6,863 participants who completed at least one cognitive test (*n* = 7,495) and one measure of social support (*n* = 7,908) from Phase 5 to 9 and had data on other covariates. Included participants tended to be younger, male and white, better educated, and employed in higher grades than those not included. Over the 10-year follow-up, 368 (5.4%) had died and 680 (9.9%) did not respond or had withdrawn from the study sample. Social support measures did not vary by participation status (*p* value range .21 to .91). Participants who dropped out were likely to have low baseline cognitive scores (*p* < .001). Table 1 displays *Z*-scores of cognition and social support. The study sample had a mean age of 55.8 years at analysis baseline. The majority were white males, 41% had a high university qualification and 43% had a high employment grade. Although half reported longstanding illness, only 13% were depressed and had a diagnosed chronic disease. Nearly 70% participants remained married during follow-up.

**Table 1.** Descriptive Statistics for Variables Used in the Analysis (*n* = 6,863)

	Measurement occasion		
	Phase 5	Phase 7	Phase 9
Variable	1997–1999	2002–2004	2007–2009
Executive function^a^			
*N*	5,970	6,324	6,045
Mean (*SD*)	0.00 (1.00)	−0.28 (0.96)	−0.36 (0.94)
Memory^a^			
*N*	6,017	6,349	6,060
Mean (*SD*)	0.00 (1.00)	−0.03 (0.99)	−0.26 (0.94)
Confiding support^a^			
*N*	6,954	6,631	6,475
Mean (*SD*)	0.00 (1.00)	0.04 (1.00)	0.10 (1.01)
Practical support^a^			
*N*	6,979	6,633	6,472
Mean (*SD*)	0.00 (1.00)	−0.06 (0.99)	−0.15 (0.99)
Negative aspects of close relationships^a^			
*N*	6,964	6,624	6,478
Mean (*SD*)	0.00 (1.00)	−0.11 (0.97)	−0.14 (0.95)
Mean age, years (*SD*)	55.86 (6.03)		
Male (%)	70.8		
White (%)	92.3		
University qualification (%)	41.0		
High employment grade (%)	42.8		
Had longstanding illness (%)	49.8		
Had depressive symptoms (%)	13.0		
Prevalent chronic disease^b^ (%)	13.9		
Always married^c^ (%)	69.9		

*Note:* ^a^All measures of cognitive function and social support were Z-scored based on Phase 5 means and standard deviations. ^b^Prevalent chronic disease was diagnosed coronary heart disease, stroke, diabetes, or cancer. ^c^Always married were those who remained married over Phases 5–9.

Table 2 gives the statistical comparison between alternative BDCSMs. For executive function and confiding support, results from goodness-of-fit indices indicated that health selection was the preferred model $(\gamma_{c\to s}=-0.11,\;$ 95% confidence interval [CI] = −0.22, −0.01). There was no evidence suggesting confiding support had effect on executive function $\text{(}\gamma_{s\to c}=-0.05,$ 95% CI = −0.17, 0.07). As such, constraining the lagged influence from confiding support to changes in executive function to zero was not associated with loss in model fit, corrected Δ χ^2^ (1, *n* = 6,859) = 0.61, *p* = .44, whereas the other models resulted in significant loss of fit. Likewise, the health selection model was also preferred for the dynamics between executive function and practical support, with a leading effect from executive function on subsequent changes in practical support ($r_{c\to s}$ = 0.18, 95% CI = 0.06, 0.30). The coupling parameters between executive function and negative aspects of close relationships were virtually zero (γ for both directions −0.04, 95% CI = −0.22, 0.14); thus, no coupling model was preferred, corrected Δ χ^2^ (2, *n* = 6,862) = 0.36, *p* = .84. The preferred models were similar for memory and social support, namely, health selection for confiding and practical support and no coupling for negative aspects of close relationships (lower part of Table 2).

**Table 2.** Comparisons of Bivariate Dual Change Score Models for Social Support and Cognition

Models					Goodness-of-fit indices					
	β _*c*_	β _*s*_	γ _*c*–*s*_	γ _*s*–*c*_	Corrected χ^2^	*p* for Lr test	BIC	TLI	CFI	RMSEA
(a) Executive function										
Confiding support										
Full coupling	−0.53 (0.03)	−0.37 (0.12)	−0.11 (0.05)	−0.05 (0.06)	−37,637.3	—	75,522	0.99	1.00	0.02
Social causation	−0.53 (0.03)	−0.30 (0.12)	0^a^	−0.05 (0.06)	−37,639.4	.05	75,517	0.99	1.00	0.02
Health selection	−0.52 (0.03)	−0.37 (0.12)	−0.11 (0.05)	0^a^	−37,637.7	.44	75,514	0.99	0.99	0.02
No coupling	−0.52 (0.03)	−0.30 (0.12)	0^a^	0^a^	−37,639.7	.12	75,509	0.99	0.99	0.02
Practical support										
Full coupling	−0.51 (0.04)	−0.36 (0.14)	0.18 (0.06)	−0.12 (0.08)	−38,379.2	—	77,006	0.99	1.00	0.02
Social causation	−0.50 (0.04)	−0.19 (0.16)	0^a^	−0.13 (0.09)	−38,383.3	.004	77,005	0.99	0.99	0.02
Health selection	−0.52 (0.03)	−0.34 (0.14)	0.18 (0.06)	0^a^	−38,381.0	.12	77,000	0.99	1.00	0.02
No coupling	−0.52 (0.03)	−0.18 (0.16)	0^a^	0^a^	−38,385.0	.009	77,000	0.99	0.99	0.01
Negative aspects of close relationships										
Full coupling	−0.51 (0.05)	−0.44 (0.22)	−0.04 (0.09)	−0.04 (0.09)	−38,446.8	—	77,141	0.99	1.00	0.02
Social causation	−0.51 (0.05)	−0.49 (0.14)	0^a^	−0.04 (0.09)	−38,446.9	.69	77,132	0.99	1.00	0.01
Health selection	−0.52 (0.03)	−0.44 (0.22)	−0.04 (0.09)	0^a^	−38,446.9	.68	77,132	0.99	1.00	0.01
No coupling	−0.52 (0.03)	−0.49 (0.14)	0^a^	0^a^	−38,447.0	.84	77,124	0.99	1.00	0.02
(b) Memory										
Confiding support										
Full coupling	−0.06 (0.01)	−0.38 (0.13)	−0.22 (0.11)	0.00 (0.01)	−43,882.9	—	87,978	0.99	0.98	0.04
Social causation	−0.06 (0.01)	−0.31 (0.12)	0^a^	0.00 (0.01)	−49,291.7	.07	87,973	0.98	0.99	0.03
Health selection	−0.06 (0.01)	−0.38 (0.13)	−0.22 (0.11)	0^a^	−43,883.0	.77	87,969	0.99	0.99	0.03
No coupling	−0.06 (0.01)	−0.31 (0.12)	0^a^	0^a^	−43,884.9	.17	87,964	0.98	0.99	0.03
Practical support										
Full coupling	−0.06 (0.01)	−0.39 (0.14)	0.40 (0.13)	−0.00 (0.01)	−44,626.7	—	89,465	0.97	0.98	0.04
Social causation	−0.06 (0.01)	−0.19 (0.16)	0^a^	−0.00 (0.01)	−44,631.3	.003	89,466	0.97	0.98	0.04
Health selection	−0.06 (0.01)	−0.38 (0.14)	0.39 (0.13)	0^a^	−44,626.9	.57	89,457	0.98	0.98	0.04
No coupling	−0.06 (0.01)	−0.19 (0.16)	0^a^	0^a^	−44,631.3	.07	89,457	0.98	0.98	0.04
Negative aspects of close relationships										
Full coupling	−0.06 (0.01)	−0.41 (0.21)	−0.18 (0.19)	0.01 (0.01)	−44,691.2	—	89,594	0.98	0.96	0.03
Social causation	−0.06 (0.01)	−0.51 (0.13)	0^a^	0.01 (0.01)	−44,692.0	.31	89,587	0.98	0.97	0.03
Health selection	−0.06 (0.01)	−0.42 (0.21)	−0.15 (0.18)	0^a^	−44,691.6	.41	89,586	0.98	0.97	0.03
No coupling	−0.06 (0.01)	−0.50 (0.13)	0^a^	0^a^	−44,692.2	.43	89,579	0.98	0.98	0.03

*Note:* β _*c*_, β _*s*_ = autoproportion parameter for cognitive function (C) and social relationships (S); γ _*c*–*s*_ = coupling parameter from cognition to social relationships; γ _*s*–*c*_ = coupling parameter from social relationships to cognition. BIC = Bayesian information criterion; CFI = comparative fit Index; corrected χ^2^ statistics adjusted for non-normal distribution; Lr tests = log-likelihood ratio tests, where a significant loss (*p* < .05) in model fit suggests full coupling model is better; RMSEA = root mean square error of approximation; TLI = Tucker–Lewis index. Cutoff values of 0.95 for TLI and CFI and 0.06 for RMSEA indicate a good model fit. Full coupling assumes bidirectional influence between social support and cognition (referent); social causation assumes unidirectional influence from social support to latent changes in cognition only; health selection assumes unidirectional influence from cognition to latent changes in social support; and no coupling model assumes independent latent changes between social support and cognition. All models adjusted for age and sex. Standard errors in parentheses.

^a^Parameters constrained to be zero.

Table 3 presents estimates from fully adjusted health selection models (The no-coupling associations of negative aspects of close relationships and cognition were not shown). As regards confiding support (first and second columns), negative coupling parameters from either measure of cognition indicated that preceding high levels of cognition predicted less improvement in confiding support (negative deviations from the positive mean slope), adjusted for the effects of autoregression and covariates. Similarly, high levels of cognition tended to positively influence subsequent changes in practical support (third and fourth columns). The paired change equations are (numbers in square brackets indicate deviations around mean slopes),

**Table 3.** Parameter Estimates From Fully Adjusted Health Selection Models for Social Support and Cognitive Function

Parameter		Confiding support		Executive function		Practical support		Executive function	
		β	95% CI	β	95% CI	β	95% CI	β	95% CI
Autoproportion β		−0.41**	(−0.65, −0.17)	−0.53***	(−0.59, −0.46)	−0.35**	(−0.58, −0.12)	−0.53***	(−0.59, −0.46)
Coupling γ		−0.11*	(−0.22, −0.01)	0^a^	—	0.18**	(0.06, 0.29)	0^a^	—
Initial mean $\mu_{I}^{b}$		0.16*	(0.12, 0.21)	0.59***	(0.56, 0.63)	0.21***	(0.17, 0.25)	0.59***	(0.56, 0.63)
Slope mean μs^b^		0.22***	(0.14, 0.31)	0.00	(−0.03, 0.04)	−0.08*	(−0.14, −0.01)	0.00	(−0.03, 0.04)
Initial variance $\sigma_{I}^{2}$		0.61***	(0.58, 0.64)	0.50***	(0.47, 0.51)	0.43***	(0.40, 0.46)	0.50***	(0.47, 0.51)
Slope variance $\sigma_{S}^{2}$		0.14*	(0.03, 0.24)	0.13***	(0.10, 0.16)	0.10**	(0.03, 0.16)	0.13***	(0.10, 0.16)
Correlation ρ _*I*,*S*_		0.18**		0.22***		0.09		0.22***	
ρ*I*_*s*_,*I*_*c*_	ρ*s*_*s*_,*s*_*c*_	−0.02		0.02		−0.02**		−0.05**	
ρ*I*_*s*_,*S*_*c*_	ρ*s*_*s*_,*I*_*c*_	−0.01*		0.06*		−0.02**		−0.09**	
Error variance ψ		0.32***		0.11***		0.40***		0.11***	

Parameter		Confiding support		Memory		Practical support		Memory	
Autoproportion β		−0.41**	(−0.65, −0.18)	−0.05***	(−0.08, −0.02)	−0.38**	(−0.62, −0.15)	−0.04***	(−0.08, −0.01)
Coupling γ		−0.22*	(−0.45, −0.01)	0^a^	—	0.39**	(0.15, 0.65)	0^a^	—
Initial mean $\mu_{I}^{b}$		0.17***	(0.12, 0.21)	0.26***	(0.22, 0.31)	0.21***	(0.17, 0.25)	0.27***	(0.23, 0.31)
Slope mean μs^b^		0.21***	(0.13, 0.29)	−0.16***	(−0.19, −0.14)	−0.06*	(−0.13, −0.00)	−0.17***	(−0.19, −0.14)
Initial variance $\sigma_{I}^{2}$		0.61***	(0.58, 0.64)	0.36***	(0.33, 0.39)	0.43***	(0.40, 0.46)	0.35***	(0.32, 0.39)
Slope variance $\sigma_{S}^{2}$		0.15*	(0.03, 0.27)	0^c^	—	0.14**	(0.04, 0.24)	0^c^	—
Correlation ρ _*I*,*S*_		0.19**		0^c^		0.10*		0^c^	
ρ*I*_*s*_,*I*_*c*_	ρ*s*_*s*_,*s*_*c*_	0.00		0^c^		−0.01		0^c^	
ρ*I*_*s*_,*S*_*c*_	ρ*s*_*s*_,*I*_*c*_	0^c^		0.08		0^c^		−0.14**	
Error variance ψ		0.32***		0.51***		0.40***		0.51***	

*Note:* 95% CI = 95% confidence interval. Autoproportion β is the self-feedback effect from variables’ preceding value; coupling γ is the lead-lag effect of one variable on subsequent change in the other; *I*_*s*_, *I*_*c*_ are intercepts for support or cognition; *S*_*s*_, *S*_*c*_ are slopes for support or cognition.

^a^Constrained to be zero as no statistically significant effects from any measure of social support to subsequent changes in cognition was found. ^b^Intercepts and slopes were conditional for 55 years, white male, with a university qualification, a high employment grade, had no longstanding illness or chronic disease, and were not depressed at Phase 5, and remained married over Phase 5 to Phase 9. ^c^Random effect was not identified for memory slope.

*p < .05, **p < .01, ***p < .001.

$$\begin{array}{l} \Delta Confiding{\left(t\right)}_{n}=\;0.22\pm [0.37]-0.41Confiding{\left(t-1\right)}_{n}-0.11Executive{\left(t-1\right)}_{n}\\ \Delta Executive{\left(t\right)}_{n}\;=\;0.00\pm [0.36]-0.53Executive{\left(t-1\right)}_{n},\\ \Delta Confiding{\left(t\right)}_{n}=\;0.21\pm [0.39]-0.41Confiding{\left(t-1\right)}_{n}-0.22Memory{\left(t-1\right)}_{n}\\ \Delta Memory{\left(t\right)}_{n}=-0.16\pm [0.00]-0.05Memory{\left(t-1\right)}_{n},\\ \Delta Practical{\left(t\right)}_{n}=\;-0.08\pm [0.32]-0.35Practical{\left(t-1\right)}_{n}+0.18Executive{\left(t-1\right)}_{n}\\ \Delta Executive{\left(t\right)}_{n}\;=\;0.00\pm [0.36]-0.53Executive{\left(t-1\right)}_{n},\\ \Delta Practical{\left(t\right)}_{n}=-0.06\pm [0.37]-0.38Practical{\left(t-1\right)}_{n}+0.39\;Memory{\left(t-1\right)}_{n}\\ \Delta Memory{\left(t\right)}_{n}\;=-0.17\pm [0.00]-0.04Memory{\left(t-1\right)}_{n}. \end{array}$$

These equations represent 5-year changes in each variable as a function of itself and the other variable conditional on other covariates included. For instance, regarding the first pair of equations, confiding support was expected to increase by 0.22 (±0.37) units over the next 5 years. This increase was decelerated if the previous level of confiding support (−0.41) or executive function (−0.11) is high. On the other hand, executive function was expected to change by 0.00 (±0.36) on average, which was negatively influenced by preceding cognitive level (−0.53) only.

**Figure 2.**
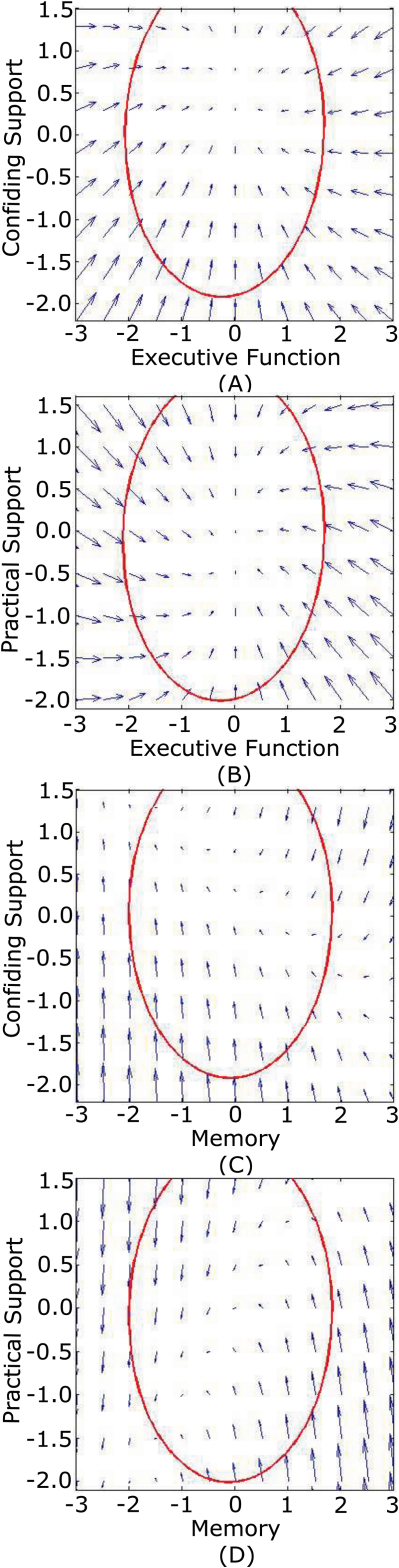
Vector fields for each bivariate dynamic system between social support and executive function (**A** and **B**) and memory (**C** and **B**) in the Whitehall II cohort. The ellipsoid encompasses 95% of the data.

To visualize the expected changes in theses bivariate systems, vector fields (Boker & McArdle, 1995) were plotted to jointly interpret these changes alongside correlations between intercepts and slopes. A given pair of coordinates is a bivariate starting point, and the directional arrow is a display of the expected pair of 5-year changes from this point (McArdle & Grimm, 2010). As illustrated in Figure 2A and C, participants with initial low levels of confiding support and cognition, perceived evident increases in confiding support. These increases were more gradual for those with higher starting levels of confiding support (*y*-axis) or higher preceding levels of executive function (*x*-axis). Changes in executive function, however, only depended on its own initial scores. As for practical support and cognition (Figure 2B and D), participants who started with higher levels of practical support were more likely to decline (*y*-axis), while higher preceding levels of cognition (*x*-axis) decelerated these processes. Changes in cognition again were not affected by levels of practical support.

Virtually identical estimates for the dynamic associations were obtained from the restricted subsample (*n* = 4,086) with complete data for 3 measurement occasions as well as among participants who remained married and consistently nominated their spouse as the closest person (*n* = 3,623). Similar dynamics were also shown in sex- and age-stratified analyses, indicating sex- and age-invariant dynamic associations (Supplementary Appendix 1 and 2).

## Discussion

We investigated the bidirectional relationships between three aspects of social support and cognition over a 10-year follow-up from middle to early old age. Our findings show that a better cognition at preceding stage was related to less positive changes in confiding support and less negative changes in practical support over the next 5 years, whereas there was no detectable influence from either measure of social support on subsequent changes in cognition. For negative aspects of close relationships, this study did not find directional relationships either to or from cognition.

Our findings endorse health selection process between cognition and social support as in some studies (Green, Rebok, & Lyketsos, 2008; Stoykova et al., 2011; Thomas, 2011), but not social causation (Ellwardt et al., 2013) or bidirectional associations (Li & Zhang, 2015) suggested by others. The current study further indicates that the influence of cognitive function on subsequent changes in social support varied by support type and the initial level of social support. The positive influence from preceding cognition on change in practical support may reflect the accessibility of practical support. Better cognition may facilitate individuals to build a strong social bank (Antonucci, Fiori, Birditt, & Jackey, 2010), the credit of which can be redeemed to fulfill their needs for support (Lang et al., 2009), presenting better maintenance when the initial level of practical support was high, or more rapid response to the needs when the initial level of practical support was low. On the other hand, the less positive improvement in confiding support may be partially due to the already high initial levels of confiding support for those participants with better cognition to begin with, who may also be more aware of the vicissitudes of life and be sensitive to negative interpersonal exchanges (Bourne, Fox, Starr, Deary, & Whalley, 2007; Staudinger, Dörner, & Mickler, 2005).

A previous study using the Whitehall II cohort examined social support over midlife (age range 37–68 years) as explanatory factors for variations in subsequent cognitive decline (Liao et al., 2014). We found that midlife cumulative high level of negative support was associated with faster decline in executive function, with a 10-year change accelerated by −0.04 *SD* (95% CI = −0.08, −0.01). In the present analysis, we allowed both social support and cognition to change from mid to late life (age range 45–80 years) and investigated the reciprocity of these dynamics. There was no evidence that differences in negative support affected 5-year changes in executive function (−0.04, 95% CI = −0.22, 0.14), although the point estimate suggested an adverse influence of negative support on cognitive decline as in the earlier study. Addressing different specific research questions, these two sets of analyses are not directly comparable. Given the modest effect size estimated previously (Liao et al., 2014) and the wide CI obtained here, it is plausible that current analysis lacked power to identify the weak effect of negative support on cognitive decline, which may also require longer time to demonstrate. Meanwhile, in light of the socioemotional selectivity theory (Carstensen et al., 2003), the quality of interpersonal relationships improves as people age. Findings from these two studies may indicate that the pernicious effects of mid-age high negative support on cognitive aging may be mitigated once the age-related decline in negative support was taken into account.

The current study is the only analysis that applied BDCSM in the context of social support and cognitive decline. By using BDCSM, this study rigorously evaluated alternative hypotheses, accounting for measurement error and systematic growth of both variables. The coupling effects of cognition on subsequent changes in social support represent deviations from the systematic growth (Lövdén, Ghisletta, & Lindenberger, 2005), above and beyond autoproportional effect and covariates included. Existing literature using cross-lagged regression (Li & Zhang, 2015) or PLCM (Ellwardt et al., 2013) cannot fully capture these dynamic features available in BDCSM. Based on different change functions, comparison between alternative statistical techniques is not straightforward (Ferrer & McArdle, 2003; Grimm, 2007). BDCSM used in our study advances other statistical models in investigating hypotheses involving dynamics and growth (Bollen & Curran, 2006; Ferrer & McArdle, 2010; Ferrer et al., 2007; Lövdén, Bergman, Adolfsson, Lindenberger, & Nilsson, 2005; McArdle, 2009).

Furthermore, although previous studies assessed the diversity of social network (Li & Zhang, 2015) or social engagement (e.g., volunteering, religious services; Thomas, 2011), our study examined the quality of support in relation to cognitive aging. It is likely that productive social interaction rather than receptive social support is cognitively stimulating (Park, Gutchess, Meade, & Stine-Morrow, 2007). We measured social support using the self-reported Close Persons Questionnaire, which evaluates the “perceived received quality of support” from close relationships based on past experiences (Stansfeld & Marmot, 1992). Being weakly associated with support actually received (Bolger, Zuckerman, & Kessler, 2000), perceived support reflects a combination of truth, personality (Bourne et al., 2007; Zammit, Starr, Johnson, & Deary, 2014), and other relational schemas (Reis, Clark, & Holmes, 2004). Close relationships, being central to people’s life, tend to be associated with more responsibilities than other peripheral social ties (Reis et al., 2004). Our findings hence may reflect different expectation in support responsiveness (Gray, 2009).

Several limitations of our study should be considered. First, our findings were based on the Whitehall II occupational cohort of British civil servants, which is not representative of the general population. Nevertheless, the level of social support (Stafford, McMunn, Zaninotto, & Nazroo, 2011) and age-related cognitive decline (Allerhand, Gale, & Deary, 2014) were comparable between our sample and the English Longitudinal Study of Ageing. Further, as the analytic sample comprises relatively healthy and younger-old persons, the extent to which observed associations are affected by dementia cases is likely to be marginal (only 57 participants had mini-mental state examination < 23 over the follow-up). Second, in this aging cohort, selective attrition is associated with low cognition but not with social support. Such nonrandom dropout dependent on cognitive competency may reduce statistical power to detect the influence of social support on the limited range of cognitive changes. That is, attrition was associated with a greater reduction in the variance of cognition than in the variance of social support, which may result in the leading effect of cognition over social support. This speculation needs to be verified by joint models that deal with missing not at random (Enders & Bandalos, 2001).

Third, in constructing the latent change equations, it is necessary to model the autoregression process. This approach however may introduce bias analogous to baseline adjustment. Although the DCSM estimates the true latent change scores free from measurement error (McArdle, 2009), we cannot entirely exclude the possibility that other unmeasured common causes associated with both baseline measures and change scores may bias the estimations obtained (Glymour, Weuve, Berkman, Kawachi, & Robins, 2005). Nevertheless, potential bias, due to baseline adjustment, is likely to be negligible for the associations of interest because the cross-sectional associations between social support and cognition were nonsignificant (*p* value range .15 to .77). Fourth, the current longitudinal panel included only three measurement occasions having both social support and cognitive measures available in the Whitehall II cohort. The wide CI around the coupling parameter suggests our study may be underpowered to detect some effects of interest. Additional phases would increase the power and gain statistical precision and facilitate the investigation on discontinuities in dynamics (Ferrer et al., 2007). Last, most of the covariates were time invariant because allowing them to vary would substantially increase modeling complexity. Moreover, change in some covariates, for example, depressive symptoms, could be direct consequences of the previous status of social support or cognition, and hence should be considered as mediators rather than as confounders.

Our study systematically investigated alternative dynamic hypotheses between social support and cognition from middle to early old age using BDCSM. Our findings suggest the health selection process, namely, cognition modified subsequent 5-year change in confiding and practical support, but not vice versa. This study contributes to the understanding of the interplay between psychosocial and cognitive aging.

## Supplementary Material

Please visit the article online at http://gerontologist.oxfordjournals.org/ to view supplementary material.

## Funding

The Whitehall II study is supported by grants from the Medical Research Council (grant K013351), the British Heart Foundation (grant RG/13/2/30098), and the National Institute on Aging, U.S. National Institutes of Health (grant AG13196). J. Head was partially supported by the Economic and Social Research Council (grant ES/K01336X/1) and the National Institute on Aging (grant R01AG013196). The funding bodies did not play any role in the study design; the collection, analysis, and interpretation of the data; the writing of the manuscript; or the decision to submit the paper for publication.

## Conflict of Interest

There is no conflict of interest to declare.

## Supplementary Material

Supplementary_Appendix
