# Supplementary material for: Dynamic Longitudinal Associations Between Social Support and Cognitive Function: A Prospective Investigation of the Directionality of Associations
Source: J Gerontol B Psychol Sci Soc Sci. 2016 Nov 1;73(7):1233–43. doi: 10.1093/geronb/gbw135 (PMC6146756; doi:10.1093/geronb/gbw135)
Supplement: Supplementary_Appendix [file gbw135_suppl_supplementary_appendix.docx]

**Appendix 1 Dynamic associations between social support and cognitive function by sex**

|  |  | **Men** | | | | **Women** | | | |
| --- | --- | --- | --- | --- | --- | --- | --- | --- | --- |
| **Parameter** |  | **N = 4859^a^** | | | | **N = 2003^a^** | | | |
|  |  | **Beta** | **95%CI** | **Beta** | **95%CI** | **Beta** | **95%CI** | **Beta** | **95%CI** |
| **Social support vs Executive Function** | | | | | | |  |  |  |
|  |  | **Confiding Support** | | **Executive Function** | | **Confiding Support** | | **Executive Function** | |
| Auto-proportion β | | -0.51*** | (-0.76,-0.29) | -0.50*** | (-0.59,-0.42) | -0.02 | (-1.00,0.97) | -0.61*** | (-0.74,-0.49) |
| Coupling γ | | -0.15* | (-0.28,-0.02) | 0.00 | (-0.15,0.14) | -0.17 | (-0.46,0.12) | 0.03 | (-0.30,0.36) |
|  |  | **Practical Support** | | **Executive Function** | | **Practical Support** | | **Executive Function** | |
| Auto-proportion β | | -0.33* | (-0.61,-0.05) | -0.50*** | (-0.57,-0.42) | -0.41* | (-0.80,-0.02) | -0.61*** | (-0.73,-0.48) |
| Coupling γ | | 0.28*** | (0.15,0.42) | -0.05 | (-0.19,0.10) | -0.08 | (-0.32,0.17) | -0.03 | (-0.21,0.16) |
|  |  | **Negative Support** | | **Executive Function** | | **Negative Support** | | **Executive Function** | |
| Auto-proportion β | | -0.21 | (-0.82,0.41) | -0.54*** | (-0.68,-0.41) | -0.54 | (-1.11,0.03) | -0.60*** | (-0.72,-0.47) |
| Coupling γ | | -0.18 | (-0.44,0.08) | 0.11 | (-0.17,0.38) | 0.09 | (-0.17,0.34) | -0.07 | (-0.30,0.17) |
| **Social support vs Memory** | | | | |  |  |  |  |  |
|  |  | **Confiding Support** | | **Memory** | | **Confiding Support** | | **Memory** | |
| Auto-proportion β | | -0.50*** | (-0.76,-0.24) | -0.02 | (-0.06,0.02) | 0.03 | (-1.03,1.09) | -0.11*** | (-0.16,-0.05) |
| Coupling γ | | -0.26* | (-0.55,0.02) | -0.01 | (-0.03,0.02) | -0.67 | (-1.74,0.40) | 0.03 | (-0.02,0.07) |
|  |  | **Practical Support** | | **Memory** | | **Practical Support** | | **Memory** | |
| Auto-proportion β | | -0.38** | (-0.65,-0.10) | -0.02 | (-0.06,0.02) | -0.37 | (-0.81,0.07) | -0.11*** | (-0.16,-0.05) |
| Coupling γ | | 0.61*** | (0.34,0.89) | -0.02 | (-0.05,0.01) | -0.33 | (-1.00,0.33) | 0.01 | (-0.05,0.07) |
|  |  | **Negative Support** | | **Memory** | | **Negative Support** | | **Memory** | |
| Auto-proportion β | | -0.18 | (-0.82,0.46) | -0.02 | (-0.06,0.02) | -0.58* | (-1.10,-0.06) | -0.10*** | (-0.16,-0.05) |
| Coupling γ | | -0.44 | (-0.99,0.11) | 0.02 | 0(-0.01,0.05) | 0.25 | (-0.32,0.81) | -0.02 | (-0.07,0.04) |
| ^a^ Numbers for confiding support were men:4858, women: 2001; for practical support (vs memory) was 4858 for men. | | | | | | | | | |
| Beta: point estimate from bivariate dual change score models, adjusted for age (centred at 55), ethnicity, education, employment grade, longstanding illness, depressive symptoms and chronic disease at phase 5 and marital history from phase 5-9. 95%CI: 95% confidence interval, * P<0.05, ** P<0.01, ***P<0.001 | | | | | | | | | |
|  |  |  |  |  |  |  |  |  |  |

**Appendix 2 Dynamic associations between social support and cognitive function by age group**

|  |  | **Age group ≤ 55 at first assessment** | | | | **Age group > 55 at first assessment** | | | |
| --- | --- | --- | --- | --- | --- | --- | --- | --- | --- |
| **Parameter** |  | **N = 3360** | | | | **N = 3502 ^a^** | | | |
|  |  | **Beta** | **95%CI** | **Beta** | **95%CI** | **Beta** | **95%CI** | **Beta** | **95%CI** |
| **Social support vs Executive Function** | | | | | |  |  |  |  |
|  |  | **Confiding support** | | **Executive Function** | | **Confiding support** | | **Executive Function** | |
| Auto-proportion β |  | -0.51*** | (-0.80,-0.22) | -0.69*** | (-0.77,-0.61) | -0.14 | (-0.65,0.37) | -0.40*** | (-0.50,-0.30) |
| Coupling γ | | -0.12 | (-0.26,0.03) | -0.07 | (-0.21,0.06) | -0.09 | (-0.26,0.08) | 0.05 | (-0.20,0.29) |
|  |  | **Practical support** | | **Executive Function** | | **Practical support** | | **Executive Function** | |
| Auto-proportion β |  | -0.40** | (-0.69,-0.11) | -0.68*** | (-0.76,-0.58) | -0.28 | (-0.71,0.16) | -0.40*** | (-0.50,-0.31) |
| Coupling γ | | 0.14 | (-0.03,0.32) | -0.03 | (-0.16,0.09) | 0.21* | (0.04,0.37) | 0.00 | (-0.21,0.20) |
|  |  | **Negative support** | | **Executive Function** | | **Negative support** | | **Executive Function** | |
| Auto-proportion β |  | -0.78*** | (-1.13,-0.43) | -0.68*** | (-0.77,-0.58) | 0.05 | (-0.90,1.01) | -0.38*** | (-0.54,-0.22) |
| Coupling γ | | 0.13 | (-0.05,0.31) | -0.01 | (-0.16,0.15) | -0.30 | (-0.67,0.08) | -0.06 | (-0.35,0.24) |
| **Social support vs Memory** | | | | | |  |  |  |  |
|  |  | **Confiding Support** | | **Memory** | | **Confiding Support** | | **Memory** | |
| Auto-proportion β |  | -0.58*** | (-0.86,-0.30) | -0.05* | (-0.09,-0.01) | -0.13 | (-0.62,0.37) | -0.05 | (-0.10,0.01) |
| Coupling γ | | -0.35* | (-0.69,-0.01) | -0.00 | (-0.03,0.03) | -0.12 | (-0.51,0.28) | 0.00 | (-0.03,0.03) |
|  |  | **Practical Support** | | **Memory** | | **Practical Support** | | **Memory** | |
| Auto-proportion β |  | -0.43** | (-0.73,-0.12) | -0.05* | (-0.09,-0.01) | -0.35 | (-0.78,0.08) | -0.04 | (-0.09,0.02) |
| Coupling γ | | 0.36 | (-0.09,0.82) | -0.02 | (-0.06,0.02) | 0.49** | (0.15,0.84) | -0.01 | (-0.05,0.03) |
|  |  | **Negative Support** | | **Memory** | | **Negative Support** | | **Memory** | |
| Auto-proportion β |  | -0.74*** | (-1.11,-0.38) | -0.05** | (-0.09,-0.01) | -0.09 | (-0.85,0.67) | -0.04 | (-0.10,0.01) |
| Coupling γ | | 0.19 | (-0.23,0.61) | 0.03 | (-0.01,0.07) | -0.39 | (-0.98,0.20) | -0.02 | (-0.06,0.01) |
| ^a^ Numbers for Age group > 55 were, 3499 for confiding support, and 3501 for practical support (in relation with memory). | | | | | | | | | |
| Beta: point estimate from bivariate dual change score models, adjusted for age (centred at 51y for age group ≤ 55, at 61 y for age group > 55), sex, ethnicity, education, employment grade, longstanding illness, depressive symptoms and chronic disease at phase 5, and marital history from phase 5-9.  95%CI: 95% confidence interval, * P<0.05, ** P<0.01, ***P<0.001 | | | | | | | | | |
